# Propolis characterization and antimicrobial activities against *Staphylococcus aureus* and *Candida albicans*: A review

**DOI:** 10.1016/j.sjbs.2021.11.063

**Published:** 2021-12-03

**Authors:** Sarra Bouchelaghem

**Affiliations:** Department of General and Environmental Microbiology, Institute of Biology, University of Pécs, Ifjúság str. 6, 7624 Pécs, Hungary

**Keywords:** Herbal product, Propolis, Flavonoids, Chrysin, Antibacterial, Antifungal, Mode of action

## Abstract

Propolis is a plant-based sticky substance that is produced by honeybees. It has been used traditionally by ancient civilizations as a folk medicine, and is known to have many pharmaceutical properties including antioxidant, antibacterial, antifungal, anti-inflammatory, antiviral, and antitumour effects. Worldwide, researchers are still studying the complex composition of propolis to unveil its biological potential, and especially its antimicrobial activity against a variety of multidrug-resistant microorganisms. This review explores scientific reports published during the last decade on the characterization of different types of propolis, and evaluates their antimicrobial activities against *Staphylococcus aureus* and *Candida albicans*. Propolis can be divided into different types depending on their chemical composition and physical properties associated with geographic origin and plant sources. Flavonoids, phenols, diterpenes, and aliphatic compounds are the main chemicals that characterize the different types of propolis (Poplar, Brazilian, and Mediterranean), and are responsible for their antimicrobial activity. The extracts of most types of propolis showed greater antibacterial activity against Gram-positive bacteria: particularly on *S. aureus,* as well as on *C. albicans*, as compared to Gram-negative pathogens. Propolis acts either by directly interacting with the microbial cells or by stimulating the immune system of the host cells. Some studies have suggested that structural damage to the microorganisms is a possible mechanism by which propolis exhibits its antimicrobial activity. However, the mechanism of action of propolis is still unclear, due to the synergistic interaction of the ingredients of propolis, and this natural substance has multi-target activity in the cell. The broad-spectrum biological potentials of propolis present it as an ideal candidate for the development of new, potent, and cost-effective antimicrobial agents.

## 1 Introduction

*Staphylococcus aureus* is a Gram-positive bacterium that is often found in the respiratory tract and the skin, while *Candida albicans* is mostly detected in the mucous membranes and in the gastrointestinal tract (Lee et al., 2019). *S. aureus* and *C. albicans* are ubiquitous opportunistic pathogens and important nosocomial strains that can cause mild to severe illnesses (Todd and Peters, 2019). The widespread use of antimicrobial drugs and the ability of certain microbes to acquire accessory genes that can cause diversity in microbes’ phenotype and resistance mechanisms has led to an unprecedented crisis of antimicrobial resistance (Aslam et al., 2018). Besides this, multidrug-resistance-carrying superbugs have increased overall mortality and morbidity rates from such infections several-fold (Fair and Tor, 2014, Frieri et al., 2017). Microorganisms acquire antimicrobial resistance by means of several underlying mechanisms, including the synthesis of enzymes that degrade the active part of antibiotics, drug efflux, modifying antibiotic binding sites, and biofilm formation (Munita and Arias, 2016). *S. aureus* and *C. albicans* have been found to form persistent biofilms on abiotic surfaces or within a host. The interaction between these biofilms is a precursor to increased drug tolerance, immune evasion, and virulence, with the outcome of this being increased mortality (Todd and Peters, 2019). For the last few decades, scientific communities have been in search of new, cost-effective, and potent antimicrobial agents to treat infections caused by multidrug-resistant strains (Aslam et al., 2018).

Natural plant-based products and synthetic chemistry are two main fields to which scientific attention has shifted in the quest to develop potent antimicrobial agents to treat and prevent infectious diseases (Abreu et al., 2012, Anand et al., 2019). Propolis is produced from the balsamic secretions of the flowers, branches, shells, leaves, barks, and buds of various plants. Honeybees (*Apis mellifera*) extract and transform this sticky substance by aid of their salivary secretions and beeswax into propolis (Elnakady et al., 2017). Propolis protects hives from moisture and predators, sealing cracks, and keeps the inner temperature of the hive warm. Since ancient times, propolis has been used as a traditional folk medicine, alone or in combination with other natural substances, to treat wounds (Rojczyk et al., 2020). The literature makes evident that propolis possesses several biological properties, including antibacterial, antiviral, antiprotozoal, antifungal, anticancer, antioxidant, antitumour, and antimutagenic activities (Elnakady et al., 2017, Ezzat et al., 2019, Kujumgiev et al., 1999, Silva et al., 2019). Several scientific reports have been published on the cytotoxicity, antioxidant and antimicrobial potential of different types of propolis (de Marco et al., 2017, Lopez et al., 2015, Mello and Hubinger, 2012). Free-radical scavenging and antimicrobial activities have presented propolis as an ideal food preservative and supplement in various food industries (Grecka and Szweda, 2021, Guzmán and Cruz, 2017). Moreover, the solvent chosen for propolis extraction can act to enhance its pharmacological potential. Ethanol extracts of propolis are more effective and show higher levels of antimicrobial activity compared to water, ester, and chloroform fractions (Wagh, 2013).

Propolis has attracted the attention of scientists searching for an alternative therapeutic drug against infectious diseases and multidrug-resistant bacteria since the 1970 s. Researchers' interest in this complex substance has increased in recent decades based on further investigation of the chemical composition of propolis (Toreti et al., 2013). However, the standardization of propolis extracts and their use in clinical treatment remains a challenge (Silva-Carvalho et al., 2015, Toreti et al., 2013). Therefore, this study seeks to review scientific reports published during the last decade on the characterization of different types of propolis around the world, their chemical composition, and to evaluate their antimicrobial activity against *S. aureus* and *C. albicans*.

## 2 Chemical composition

Propolis is currently gaining the attention of scientific communities due to its wide-ranging biological application. It is a highly complex substance, and several factors influence its chemical composition, including the plant sources surrounding beehives, honeybee species, method of collection, geographical and climatic variation, collecting seasons, altitudes, and adequate lighting (Bueno-Silva et al., 2017, López and Sawaya, 2012). Propolis is a sticky substance that contains 50% plant resins, 30% wax, 10% essential oils, 5 % pollen, and 5% other organic compounds (Brown, 1989). More than 400 compounds had been identified in poplar-type propolis by 2014 (Ristivojević et al., 2015). This list of compounds is still increasing, and the propolis samples collected from different parts of the world had revealed 850 components up to 2018 (Šturm and Ulrih, 2019). The known components of propolis are grouped into chemical classes that include: alcohols, alkans, volatile oils, aromatic acids, amino acids, vitamins, sugars and sugar alcohols, terpenoids, fatty acids, hydrocarbons, wax esters, flavonoids, chalcones, phenols, glycerol derivatives, aldehydes, trace elements, small proportions of minerals, and ketones (Ahangari et al., 2018, Šturm and Ulrih, 2019). These categories include various active compounds, such as flavones, caffeic acid, isovanillin, vanillin, butanoic acid, malic acid, alanine, benzoic acid, coumaric acid, gentisic acid, ferulic acid, vanillic acid, pinocembrin, pinobanksin, galangin, thymol, luteolin, terpenes, lignans, myricetin, decanoic acids, chrysin, quercetin, and kaempferol (Kurek-Górecka et al., 2013, Šturm and Ulrih, 2019). The therapeutic properties of propolis are mainly attributed to volatiles (Bankova et al., 2014, Jihene et al., 2018), flavonoids, and phenolic compounds which are well known as antioxidant and antimicrobial active ingredients (da Silva et al., 2006, Kurek-Górecka et al., 2013). Chrysin is a plant flavone extracted from the leaves of *Passiflora caerulea*, and it is found in honey and propolis (Mani and Natesan, 2018). The anticancer and cytotoxicity activities of propolis are related mainly to chrysin (Celińska-Janowicz et al., 2018, Seetharaman et al., 2017). Some studies report that chrysin has antimicrobial properties based on its ability to destroy the integrity of the microbial cell wall and cell membrane (Celińska-Janowicz et al., 2018, Mani and Natesan, 2018). Besides, this other polyphenols (such as caffeic acid, ferulic acid, and *p*-coumaric acid) in propolis affect DNA biosynthesis in cancer cells (Liu et al., 2014, Suresh Babu et al., 2006, Vardar-Ünlü et al., 2008). Genistein is one of the natural isoflavones detected in propolis (Gargouri et al., 2019, Volpi and Bergonzini, 2006), and is mainly found in *Glycine* max L*.* and *Trifolium* species. It has received widespread attention due to its chemotherapeutic activity against different types of cancer, mainly by altering apoptosis (Spagnuolo et al., 2015), and reduction in chronic inflammatory disorders (Vanden Braber et al., 2018). It enhances the immune response of macrophages against *C. albicans* (Cui et al., 2016), and acts as an antibacterial agent against *S. aureus* (Choi et al., 2018). Pinocembrin is one of the primary flavonoids abundant in poplar-type propolis. Its pharmacological activities have been well studied, including anti-inflammatory, antioxidant (Rasul et al., 2013) and antibacterial action against *S. aureus*, *Escherichia coli*, and *Klebsiella pneumonia* (Tundis et al., 2019), and antifungal against *Penicillium italicum* (Peng et al., 2012). Malic acid is a chemical found in fruits and used as a flavouring in drinks and foods, and has shown antimicrobial activity against a wide range of bacterial strains of *Listeria monocytogenes*, *Salmonella enteritidis* and *Escherichia coli* (Raybaudi-Massilia et al., 2009). Propolis also contains vanillin, glycerol, and glycolic acid, which are used in other fields like cosmetic products and food additives, due to their anti-aging, antimicrobial, antiviral, and antioxidant properties (Boonchird and Flegel, 1982, Talla et al., 2017). In addition, propolis comprises certain components that are still not well known to have any antimicrobial activity, including fatty acids and sugars. Propolis has long been confirmed as an interesting pharmaceutical agent: however, its biological activity is associated with the synergistic activity of many classes of its active ingredients (Kujumgiev et al., 1999).

## 3 Types of propolis

To date, several types of propolis have been identified based on chemical composition and plant origin, the most famous of which are poplar-type (Eurasian) propolis, Brazilian green and red propolis, and Mediterranean propolis (Table 1). The huge heterogenicity in the chemical composition of propolis needs to be carefully analysed to ensure that the appropriate type of propolis is used, for safer and more effective treatment. The process of standardization and homogenization is extremely challenging and requires innovative, cost-effective, and efficient technologies such as high-performance liquid chromatography (Bruschi et al., 2003, Cuesta-Rubio et al., 2007), thin-layer chromatography (Milojković-Opsenica et al., 2016), liquid chromatography and gas chromatography coupled with other powerful techniques such as mass spectrometry (Asgharpour et al., 2020, Cheng et al., 2013, Falcão et al., 2013, Popova et al., 2010), and nuclear magnetic resonance (Cuesta-Rubio et al., 2007, Kasote et al., 2017). The chemical composition of poplar-type propolis is well studied among different types of propolis and offers an ideal standardization model (Bankova, 2005). Propolis varies in colour from dark yellow, to greenish-brown, to red, due to its age and nearby plant sources, while terpenes and phenolic compounds are accountable for its distinctive scent (Devequi-Nunes et al., 2018). Using high-performance thin-layer chromatographic fingerprinting analyses to explore the chemical composition of propolis, studies have confirmed the existence of two different subtypes of European propolis, as orange and blue types (O-type and B-type), originating from *Populus nigra* and *Populus tremulas*, respectively (Degirmencioglu et al., 2019, Milojković Opsenica et al., 2016, Ristivojević et al., 2015). On the other hand, green type (G-type) propolis is distinguished by its mixture of light orange, dark green, and blue bands (Ristivojević et al., 2015). O-type propolis is characterized by quercetin, while B-type corresponds mostly to galangin, caffeic acid, feruloyl, and *p*-coumaroyl derivatives. G-type corresponds to apigenin or naringenin. However, some German propolis samples have been classified as of mixed type (Morlock et al., 2014). Brazilian propolis has been classified into 12 types, based on physical and chemical properties and geographical locations, but only three species of plant sources have been identified: namely *Populus* sp., *Hyptis divaricate,* and *Baccharis dracunculifolia* (Alencar et al., 2007, Silva et al., 2008). Green and red Brazilian propolis types are well known compared to newer types like yellow and brown propolis, which still need further characterization (Machado et al., 2016). The Mediterranean type has distinctive chemical properties, and is exceptionally rich in diterpenes and their derivatives (Popova et al., 2012).

**Table 1:** Chemical characterization of different types of propolis, geographic distribution, botanical origin, and biological activities.

Type	Region	Main compounds	Plant source	Activity	Cell used	Reference
Green propolis	Brazil Taiwan	Apigenin Artepillin C Caffeic acid Chrysin Cinnamic acid Ferulic acid Kaempferide Narigenin Pinobanksin Rutin	*Baccharis dracunculifolia Eucalyptus citriodora Araucaria angustifolia Mimosa tenuiflora*	Antibacterial Antibiofilm Antioxidant	*Bacillus Subtilisn* *Escherichia coli* *Listeria monocytogenes* MRSA MSSA *Pseudomonas aeruginosa*	(Bezerra et al., 2020, Búfalo et al., 2009, Chen et al., 2018, Corrêa et al., 2020, Ferreira et al., 2017, Roberto et al., 2016)
				Antifungal	*Candida albicans* *Candida parapsilosis* *Candida tropicalis*	
				Anti-genotoxic	*Allium cepa*	
				Antitumour	HEp-2	
Red propolis	Brazil Cuba	Artepellin C Biochanin A Flavone Homopterocarpin Liquiritigenin Lupeol Medicarpin Methyl abietate Methyl *o*-orsellinate Naringenin Neovestitol Pterocarpans Vestitol *β*-amyrin	*Dalbergia ecastophyllum Clusia* sp. (*C. scrobiculata, C. minor, C. major,* and *C. rosea*)	Antibacterial Antioxidant	*Bacillus subtilis* *Enterococus faecalis* *Enterococcus* sp. *Escherichia coli* *Klebsiella sp*. MRSA *Pseudomonas aeruginosa* *Streptococcus mutans*	(Alencar et al., 2007, Andrade et al., 2017, Cuesta-Rubio et al., 2007, Dantas Silva et al., 2017, Machado et al., 2016, Piccinelli et al., 2011, Regueira et al., 2017, Rufatto et al., 2018)
				Antiparasitic	*Trypanosoma cruzi epimastigotes Y*	
				Antitumour	HCT-116 SF-295 HL-60 OVCAR-8	
Brown propolis	Brazil Cuba	Artepillin C Baccharin Caffeic acids Chlorogenic acids Drupanin Kaempferide Kaempferol *p*-coumaric Phenylpropanoid Polyisoprenylated benzophenones Prenylated phenylpropanoids	*B*. *dracunculifolia C. rosea*	Anti-mycoplasma	*Mycoplasma* sp. (*M. bovis, M. gallisepticum, M. genitalium, M. hominis, M. hyorinis, M. penetrans,* and *M. pneumonieae*)	(Andrade et al., 2017, Cuesta-Rubio et al., 2007, Dantas Silva et al., 2017, de Oliveira Dembogurski et al., 2018, do Nascimento Araújo et al., 2020, Machado et al., 2016)
				Antibacterial Antibiofilm Antioxidant	*Enterococcus* sp. *Staphylococccus aureus*	
				Antiparasitic	*Trypanosoma cruzi epimastigotes Y* *Trichomonas vaginalis*	
				Antitumour	OVCAR-8	
Mediterranean propolis	Greek Cyprus Malta Sicily Bulgaria Turkey Greece Algeria Croatia Morocco	Communic acid Diterpenic acids Hydroxyditerpenic acid Imbricataloic Isoagatholal Isocupressic acid Pimaric acid Pinocembrin	*Cupressus sempervirens Pinus species*	Antibacterial Antibiofilm Antioxidant	*Enterobacter cloacae* *Escherichia coli* *Klebsiella. pneumoniae* MRSA *Pseudomonas aeruginosa* *Staphylococcus epidermidis* *Streptococcus mutans* *Streptococcus viridans*	(El-Guendouz et al., 2016, Piccinelli et al., 2013, Popova et al., 2010, Velikova et al., 2000, Popova et al., 2012)
				Antifungal	*Candida albicans* *Candida tropicalis* *Candida glabrata*	
Yellow propolis	Cuba Brazil	Acetyl triterpenes Flavanones Lanostane Lupane Oleanane Polymethoxylated Sterols Triterpenic alcohols Ursane	Undetermined	Antibacterial	*Staphylococcus aureus*	(Cuesta-Rubio et al., 2007, Machado et al., 2016, Márquez Hernández et al., 2010, Monzote et al., 2012)
				Antifungal	*Trichophyton rubrum*	
				Antiprotozoal	*Leishmania infantum* *Plasmodium falciparum* *Trypanosoma brucei* *Trypanosoma cruzi*	
				Antitumour	MRC-5 OVCAR-8	
Poplar propolis	Mostly from Eurasian regions*	Acetyloxycaffeate Caffeic acid Chrysin Dihydroflavonols Galangin Henolics Phenylpropanoids Pinobanksin Pinocembrin Prenyl caffeate Salicylic acid	*Populus* sp. (*P. nigra* L., *P. tremuloide, and P. alba* L.)	Antifungal	*Aspergillus fumigatus* *Candida glabrata* *Candida albicans* *Fusarium* sp.	(Boisard et al., 2020, Boisard et al., 2015, de Marco et al., 2017, Dezmirean et al., 2017, Popova et al., 2007, Ristivojević et al., 2020, Vardar-Ünlü et al., 2008, Wang et al., 2014)
				Antibacterial Antibiofilm Antioxidant	*Acinetobacter baumannii* *Bacillus cereus* *Enterococus* sp. *Escherichia coli* *Lactobacillus acidophilus* *Listeria* sp. *Mycobacterium smegmatis* *Pseudomonas aeruginosa* *Salmonella enteritidis* *Staphylococcus* sp. *Streptococcus* sp.	
				Anti-inflammatory	Murine macrophage RAW 264.7 HEK-293 T and HEK-293	

(MRSA) Methicillin-Resistant *Staphylococcus aureus.* (MSSA) Methicillin-Sensitive *Staphylococcus aureus.* (HEp-2) Human epidermoid carcinoma. (HCT-116) Colorectal carcinoma. (SF-295) Human glioblastoma. (HL-60) Human leukaemia. (OVCAR-8) Human ovarian carcinoma. (MRC-5) Human simian virus 40-immortalised lung fibroblasts. (HEK-293 T and HEK-293) Human embryonic kidney cells.* England, France, Italy, Switzerland, Germany, Poland, New Zealand, Russia, Bulgaria, Macedonia, Estonia, Latvia, Lithuania, Slovakia, Slovenia, Serbia, Ukraine, Hungary, Syria, Turkey, Iran, Argentina, Canada, Chile, China, Korea, Uruguay, Uzbekistan, and the USA.

## 4 Geographic distribution and botanical origin of propolis

The literature highlights the crucial role played by geographic region in types of propolis (Table 1), mainly due to climatic variation and different ethnobotanical flora by region (Bueno-Silva et al., 2017). Poplar, alder, willow, elm, birch, horse-chestnut, beech, and conifer tree species are popular sources for the finest quality propolis (Toreti et al., 2013). *P. nigra,* commonly known as the poplar, is widely distributed in Europe and North America, Asia, and New Zealand (Dezmirean et al., 2021). Russian birch propolis collected from *Betula verrucosa* is different from poplar propolis, and comprises flavonols and flavones (Bankova, 2005). *Dalbergia ecastophyllum, Clusia scrobiculata, Clusia minor, Clusia major,* and *Clusia rosea* are the plant sources for the red propolis that is widely distributed in Brazil, Cuba, M**e**xico, China and Venezuela, and is characterized by polyisoprenylated benzophenones as active phytochemicals (Rufatto et al., 2017). Similarly, the leaf resin of *Baccharis dracunculifolia* accounts for the collection of Brazilian propolis and contains a variety of phytochemicals, including flavonoids, lignans, *p*-coumaric acid, diterpenes, acetophenone, and higher concentrations of artepillin C (Anjum et al., 2019). Certain phytochemicals such as sesquiterpenoid compounds including ledol, germacren D, and spatulenol are limited to tropical regions. Also, a Mediterranean propolis type is found in Greek, Cyprus, Croatia, Egypt, Algeria, Morocco, and Malta, whose main compounds are diterpenes most probably originating in the coniferous plant of the genus *Cupressaceae* (El-Guendouz et al., 2018, Ezzat et al., 2019, Piccinelli et al., 2013, Popova et al., 2012, Popova et al., 2010). Propolis samples from different geographic origins were investigated for their antibacterial and antifungal properties. Significantly, all the propolis samples were active against *S. aureus* and *C. albicans*, despite the great differences in the plant origins between the samples from the temperate and tropical zones (Table 1).

## 5 Antibacterial and antifungal activities

### 5.1 Anti- staphylococcal activity

Due to the development of microbial resistance against various antibiotics (Aslam et al., 2018), there has been a growing interest in identifying effective antimicrobial agents obtained from various natural products (Guzmán and Cruz, 2017). Propolis is one of the most promising sources of bioactive compounds to show antimicrobial activity (AL-Ani et al., 2018). The antibacterial potential of propolis varies considerably from one bacterial strain to another, and depending on the propolis sample used (Almuhayawi, 2020). In many scientific studies, propolis and its derivatives have shown significant antibacterial activity against *Escherichia coli*, *S. aureus*, *Streptococcus* species, *Salmonella typhi*, *Enterococcus species*, *Bacillus* species, and *Pseudomonas aeruginosa* (Anjum et al., 2019, Przybyłek and Karpiński, 2019, Rufatto et al., 2017). Literature suggests that alcohol fractions of propolis possess significant antibacterial activity against Gram-positive as compared to Gram-negative bacteria (Przybyłek and Karpiński, 2019). In Lu et al.’s (2005) study, an ethanolic extract of Taiwanese propolis showed high levels of antibacterial activity against *S. aureus* with a minimum inhibitory concentration (MIC) of lower than 3.75 to 60 µg/mL, and a minimum bactericidal concentration (MBC) which ranged between 7.5 and 120 µg/mL, hence being found to be effective. The same study confirmed the influence of season and area of the collected samples on propolis activity. In addition, the age of bacterial cells, a temperature of 37 °C, and an acidic pH enhanced the antibacterial activity of the propolis extract (Lu et al., 2005). The highest anti-staphylococcal activity levels of ethanolic extract of propolis (EEP) after Taiwanese propolis was recorded for samples collected from Turkey, Oman, and Ireland, with MIC values of 8, 42, and 80 μg/mL, respectively (AL-Ani et al., 2018, Popova et al., 2013, Uzel et al., 2005). Some Brazilian propolis samples showed a very broad range of MIC, from 31.2 µg/mL to higher than 1024 µg/mL, against *S. aureus* strains (Bueno-Silva et al., 2017, Regueira et al., 2017). An ethanolic extract of Chilean propolis inhibited the growth of Gram-positive bacteria only, and showed very weak antibacterial activity against *Streptococcus pyogenes* and *S. aureus* (ATCC 25923), with an MIC of 200–26900 µg/mL. Interestingly, the total phenolic content of Chilean propolis was not correlated with the MIC values (Bridi et al., 2015). An antibacterial study of Mediterranean propolis samples was carried out by the disc diffusion method against Gram-positive and Gram-negative bacteria and oral pathogens. It is noteworthy that the diterpene content in the EEP samples was directly proportional to antimicrobial activity against all tested bacteria. Moreover, the samples showed particularly strong activity on Gram-positive bacteria (*S. aureus, Staphylococcus epidermidis, Streptococcus mutans*) (Graikou et al., 2016). Further studies on propolis samples collected from Mediterranean areas confirmed the effectiveness of EEP on *S. epidermidis*, *S. aureus* and methicillin-resistant *S. aureus* (MRSA) using a disc diffusion assay at a concentration range of 100 to 1000 µg/mL, with an inhibitory zone of 4.6–21.4 mm (Béji-Srairi et al., 2020, Benhanifia et al., 2014, Nedji and Loucif-Ayad, 2014), and MBC values of 980 and 1220 µg/mL on *S. aureus* ATCC 6538 and MRSA strains respectively (El-Guendouz et al., 2018). Interestingly the Tunisian EEP showed strong antibacterial activity on Gram-negative bacteria (Béji-Srairi et al., 2020). Chloroform fractions of Brazilian red propolis (BRP) have shown antibacterial activities against *S. aureus* and *Streptococcus mutans,* with MIC values ranging from 25 to 50 µg/mL (Alencar et al., 2007). Another study investigated the antimicrobial potential of methanol, acetate and hexane fractions of BRP against reference strains including *S. aureus* (ATCC 13,150 and 25,923), *S. epidermides* (ATCC 12228) and *Pseudomonas aeruginosa,* showing significant antibacterial activity at MIC values ranging from 128 to 512 µg/mL (Neves et al., 2016). Similarly, ethanol extracts of Polish propolis (EEPP) have shown antibacterial activity against *S. aureus* (ATCC 25,923 and ATCC 29213) with MIC values ranging from 128 to 512 µg/mL, and weak bactericidal activity with MBC values from 512 up to 4096 µg/mL. However, *S. epidermidis* ATCC 12,228 was more susceptible at MIC and MBC values in the range of 32 and 512 µg/mL (Grecka et al., 2019). Siriwong et al. (2016) found that some propolis compounds modulated resistance to conventional antibiotics, with quercetin for example showing synergistic effects with amoxicillin and reduced resistance in amoxicillin-resistant *S. epidermidis* to *β*-lactam antibiotics (Siriwong et al., 2016).

Infections caused by biofilms are causing challenges, as eradication of biofilms with conventional antibiotics is becoming more difficult (Arciola et al., 2018). Several reports have shown that antibiotics are often ineffective in eradicating biofilms (Daikh et al., 2020). Use of ethanolic extracts of Brazilian brown propolis was investigated with mature biofilms of *S. aureus*, and the results included a reduction of 93% of the viability of the cells present in the biofilms at 125 μg/mL. However, total biofilm biomass eradication was insignificant (de Oliveira Dembogurski et al., 2018). Algerian propolis exhibited a difference in biofilm inhibition across *S. aureus* ATCC 29213, *S. aureus* ATCC 33862, and MRSA strains based on the extraction solvent used and the origin of the propolis samples. A petroleum ether extract of Algerian propolis eradicated 40–80% of 48 h-old biofilm at a concentration of 300 μg/mL (Daikh et al., 2020). At an MIC value of 360 µg/mL, Moroccan propolis extract significantly reduced the virulence of *S. aureus* ATCC 6538 and MRSA. Furthermore, continued exposure to propolis treatments did not lead to the development of bacterial resistance (El-Guendouz et al., 2018). EEPP has shown antibiofilm activity against reference strain of *S. epidermidis* ATCC 35,984 with the MBEC_50_ (minimal biofilm eradication concentration that causes a total of 50% reduction in biofilm) equivalent to an MIC value of 128 µg/mL (Grecka et al., 2020). A study by Wojtyczka and colleagues found moderate inhibition of *S. epidermidis* biofilm with 780 to 1560 µg/mL of EEP after 24 h incubation (Wojtyczka et al., 2013b). However, *S. aureus* biofilms were completely inactivated with 2 μg/mL EEP after 40 h’ treatment, indicating that activity is dependent on treatment time (Ambi et al., 2017). Grecka et al. revealed the high efficiency of EEP in the eradication of MSSA biofilms incubated for 24 h at 37 °C, with equal values of MIC and MBEC_50_ (64–128 µg/mL). It was concluded that the antibiofilm activity of propolis was its most clinically beneficial aspect (Grecka et al., 2019). The antibiofilm activity of Russian propolis ethanol extracts (RPEE) on mature biofilm has been reported by Bryan et al., using MTT assay. Their study showed a 50% decreased viability of *S. aureus* at a high concentration (5% w/v) of RPEE. However, at fairly high RPEE concentrations (20% w/v), confocal and scanning electron microscopy images indicated complete cell lysis of bacterial biofilms after 18 h treatment (Bryan et al., 2015). Generally, propolis may be an excellent candidate for combating nosocomial diseases and eradicating biofilm on medical equipment caused by *S. aureus* (El-Guendouz et al., 2018).

### 5.2 Anti-candida activity

The increasing number of fungal infections is a troublesome problem in particular for immunocompromised patients (Gucwa et al., 2018). The genus *Candida* refers to a fungus that forms part of the individual's microbiota, and is largely present in areas of mucous membrane such as the oral and vaginal cavity (Capoci et al., 2015). *Candida albicans* and other species are opportunistic pathogens which have been recorded as the most frequent cause of candidiasis (Gucwa et al., 2018) and candidemia (Mutlu Sariguzel et al., 2016). Furthermore, many hospital-acquired infections are associated with the ability of microorganisms to adhere to human cells (Capoci et al., 2015), and to form biofilms in implanted orthodontics, catheter materials, and other medical devices (Gucwa et al., 2018). Thus, the formation of biofilm by *C. albicans* is one of several virulence factors responsible for infectious disease, and increases the risk of periodontal disease (Siqueira et al., 2015), vulvovaginal candidiasis (Capoci et al., 2015), and the development of various mechanisms of resistance against antifungal agents (Bezerra et al., 2020).

Some studies have supported the importance of using natural products such as propolis to treat fungal infections caused by *Candida* species. Although the antimicrobial activity of propolis has been investigated over recent years as an alternative for conventional therapeutic strategies, the antifungal activity of propolis is still underestimated, and therefore needs more evaluation to determine its therapeutic role. An ethanolic extract of Turkish propolis showed the highest antifungal activity against 76 candida isolates (*C. albicans*, *C. parapsilosis*, *C. tropicalis*, and *C. glabrata*) that were isolated from the blood cultures of intensive care unit patients, with an MIC range of 0.185 to 3 μg/mL (Mutlu Sariguzel et al., 2016). Among 19 *Candida* species, *C. albicans*, *C. glabr*ata, and *C. tropicalis* were isolated from chronic periodontitis cases, and about 42% of *C. albicans* isolates were resistant to fluconazole. However, all *Candida* species were sensitive to alcoholic extract of BRP. Fungistatic (MIC) and minimum fungicidal concentration (MFC) activities of propolis extract on *C. albicans* were observed in the range of 32–64 μg/mL and 64–512 μg/mL, respectively (Siqueira et al., 2015). Ethanolic extract of BRP showed MIC and MFC at 256 µg/mL on all yeast cells (*C. albicans*, *C. tropicalis*, and *C. neoformans*): however, hexane, acetate, and methanol fractions of the same samples of propolis showed antifungal activity at MIC values ranging between 32 and 1024 µg/mL (Neves et al., 2016). Propolis samples collected from Tunisia exhibited intense antifungal activity against all tested *Candida* species (*C. albicans* ATCC 90028, *C. glabrata* ATCC 90030, *C. parapsilosis* ATCC 22019, and *C. krusei* ATCC 6258) at a concentration of 250 µg/mL (Béji-Srairi et al., 2020). Moreover, other studies suggest that the crude extract of any natural product displaying MIC lower than 500 µg/mL is a promising substance (Duarte et al., 2007, Tiveron et al., 2016). Different BRPs showed an MIC in the range of 250–1000 µg/mL using the serial microdilution method on *C. albicans* (López et al., 2015). Most likely, extraction method affects the activity of propolis: an ethanolic extract of French poplar-type propolis showed considerable activity against *C. albicans* at an MIC equal to 31.25 µg/mL (Boisard et al., 2015).

The ability for morphological transition between yeast cells and hyphal forms is an important virulence factor for candidiasis that is caused mainly by *C. albicans* infection. MIC and MFC values of an ethanolic extract of Iranian propolis against fluconazole-resistant *C. albicans* isolates (from nails, the oral cavity, and vaginal cavity) ranged from 120.2 to 970.6 µg/mL and 480.8 to 3900.4 µg/mL, respectively. The sub-inhibitory concentrations (1/2 MIC and 1/4 MIC) significantly reduced germ tube formation (Haghdoost et al., 2016). The MFC was in a range comparable to the fungicidal activity of BRP, observed as 64–512 μg/mL against *C. albicans* strains (Freires et al., 2016, Siqueira et al., 2015). Bezerra et al. found that the green propolis ethanolic extract showed significant antifungal activity, using disk diffusion assay, against *C. albicans* and *C. tropicalis,* with MIC values ranging from 2.5 to 250 μg/mL, while *C. parapsilosis* was found to be less sensitive. The EEP exhibited antiadhesion activity at concentrations of 2.5 and 250 μg/mL after 12 h, and highly significant antibiofilm activity (0.25–250 μg/mL) after 24 h and 48 h incubation, where a reduction of from more than 30% to 100% of colony-forming units (CFU) was observed for the three *Candida* species on surfaces of steel and acrylic resin of orthodontic material (Bezerra et al., 2020). Furthermore, propolis could be a promising anti-cariogenic agent, and has shown efficiency in reducing the CFU of *C. albicans* by between 33 and 79 % CFU in mature biofilm. Thus, propolis is considered a good oral antiseptic to prevent caries (Djais et al., 2019). Another study investigated the effect of Brazilian propolis extract in solution for anti-biofilm activity against 29 clinical isolates of *C. albicans* isolated from vaginal specimens. The EEP showed strong anti-biofilm activity against all the isolates, with MIC values ranging between 68.35 and 546.87 μg/mL, in which 75.8% of the total isolates died at a concentration of 546.87 μg/mL (Capoci et al., 2015). A study by Gucwa et al. (2018) tested EEPP on biofilms from 34 clinical isolates of three species from the *Candida* genus, using MTT assay. Most of the EEPP samples showed high antibiofilm activity, and 50% of mature biofilm of *C. albicans* was eradicated at from 81 µg/mL to more than 2540 µg/mL. In addition, the biofilms of *C. krusei* and *C. glabrata* were less resistant to propolis treatment (Gucwa et al., 2018). More than 84% inhibition was found for the morphological transformation of *C. albicans* from yeast cell to hyphal forms after 2 h exposure to subinhibitory concentrations of EEPP. Excessive use of antimicrobial drugs often leads to resistance among microorganisms: hence the need to use higher and higher doses of drugs, which can be toxic to human cells. Gucwa et al. (2018) revealed a synergistic effect between the components of propolis and antifungal drugs (fluconazole and voriconazole) against *C. albicans*. This finding could be interesting from a clinical point of view. Therefore, propolis has potential use in modifying the adhesive properties of *C. albicans*, thus preventing the pathogen’s ability to form biofilms (Feldman et al., 2014). Additionally, propolis extracts could prevent yeast cells from forming biofilms while showing very low cytotoxicity in human cells (Capoci et al., 2015).

## 6 Mechanism of action

Propolis and some of its derivatives are responsible for either killing bacterial cells directly by interacting with them through different mechanisms, or by modifying the immune response of host cells (Almuhayawi, 2020). It is evident from the literature that several possible mechanisms might account for the lower antibacterial activity of propolis against Gram-negative bacteria. One possible reason could be the synthesis of a wide variety of hydrolytic enzymes by Gram-negative microorganisms (Grecka et al., 2019). These hydrolytic enzymes may interfere with the active components of propolis and result in the development of resistance (Bryan et al., 2015). Several underlying mechanisms have been proposed by different research groups regarding the antimicrobial activity of propolis, including the inhibition of cell division, nucleic acid synthesis, protein synthesis, impeding cytoplasmic membrane function, altering membrane permeability, reducing the ability to form biofilms, bacteriolysis, inhibiting the energy generation pathway, and reducing bacterial resistance towards certain conventional antibiotics (Przybyłek and Karpiński, 2019).

The effect of propolis on the bacterial cell membrane’s integrity was assessed for *S. aureus* and *E. coli* by measuring the release of intracellular constituents into the medium. The results indicate that the ethanolic extract of Brazilian propolis causes irreversible damage to the bacterial cell membrane, leading to cell death (Torres et al., 2018). Due to the different quality, quantity, and ratios of each component of propolis, it is difficult to predict the predominant biological activity of this natural substance, as it is considered that these components act synergistically. The synergistic interaction between EEP and antibiotics on *S. aureus* and other microorganisms has been identified by the broth microdilution and disc diffusion methods, confirming the enhancement of the antimicrobial action of *β*-lactam antibiotics in the presence of propolis (Regueira Neto et al., 2017), through inhibition of *β*-lactamase enzymes and peptidoglycan synthesis. Therefore, propolis revealed synergistic interaction with antibiotics that act on inhibiting the cell wall, proteins synthesis, and ribosomes. The results further indicate that therapy with a combination of propolis and other drugs reduces the risk of developing multidrug-resistant microorganisms during treatment (Grecka et al., 2019, Grecka and Szweda, 2021, Regueira et al., 2017, Wojtyczka et al., 2013a). A study by Ambi et al evaluated the activity of Russian propolis ethanol extract (RPEE) against *S. aureus* and *E. coli*. It was detected that RPEE causes cell lysis and bacterial cell membrane damage within mature biofilms at a concentration of 2–4 μg/mL, and the authors state that the structural mechanism of action stems from antibacterial and anti-biofilm activities related to the duration of exposure to propolis (Ambi et al., 2017). It is remarkable that this propolis was found to have the ability to completely inactivate bacterial cells within the biofilm matrix after 18 h of treatment, demonstrating severe cell wall damage. Thus, the mechanism of action of RPEE is structural rather than functional (Bryan et al., 2015).

The fungal cell wall is the first barrier responsible for growth, adaptation, and permeability regulation of fungal pathogens during infection (Gucwa et al., 2018). Corrêa and colleagues found that Brazilian propolis damages the integrity of *C. albicans’* cell wall and cell membrane, and causes leakage of intracellular organelles. The study hypothesizes that the antifungal efficacy of propolis is due to the capacity of polyphenols to form a complex with soluble proteins by disrupting the synthesis of chitin, which leads to cell wall disruption (Corrêa et al., 2020). After measurement of *C. albicans* growth in the presence and absence of an osmoprotectant (sorbitol), the results revealed that ethanolic extracts of polish propolis do not affect the cell wall. However, ergosterol and membrane depolarization assays suggest that the cell membrane might be a potential target for propolis (Gucwa et al., 2018).

A study by Aru et al. found that Turkish propolis extract caused an apoptotic effect on cancer cell lines, and promoted cell cycle arrest by activating the expression of cell cycle p21 proteins. Using MTS assay, the same propolis samples showed moderate anti-proliferative activity on cancer cell lines (Aru et al., 2019). Propolis-derived antiviral activity against human rhinovirus (HRV) was evaluated using sulforhodamine B assay and real-time reverse transcription-polymerase chain reaction. The results showed a significant decrease in HRV RNA replication into human epithelial adenocarcinoma cervix (HeLa) cell cultures. Kaempferol and *p*-coumaric acid may interfere with expression of intercellular adhesion molecules (Kwon et al., 2019).

These studies indicate that the mode of action of propolis is not determined by identifying the mode of action of its bioactive constituents separately, but that it is a complex interaction between all the compounds. Nevertheless, very little is currently known about the molecular mechanisms associated with the biological effects of propolis (Boisard et al., 2020), and the mechanisms underpinning its activity against microorganisms are still not clear. However, for a long time, it has been considered that the activities of propolis compounds against microorganisms are more related to the synergistic effect of polyphenols than to individual effects (Koo et al., 2000, Martins et al., 2002).

## 7 Conclusion

Propolis is an effective natural product that offers a wide variety of biological potentials, including antimicrobial activities, in addition to other pharmaceutical applications. The chemical composition of propolis is highly complex and varies from one geographical region to another. Despite the numerous studies dealing with this highly complex substance, it is currently challenging to standardize. It is established that the type of propolis varies depending on geographical origins and plant sources, with huge heterogeneity in chemical composition. Ethanol extracts of propolis are of great significance, exhibiting higher antibacterial and antifungal activities against multidrug resistant strains. Polyphenols, terpenes, and aromatic compounds are the major phytochemicals to show remarkable antimicrobial activities, and the activity of these chemicals can be based on a single action or synergistic interaction between several components. Finally, the current review recommends further study of the biological potentials and mechanisms of action of new types of propolis from diverse regions, for the prevention and control of human infectious diseases.

## Declaration of Competing Interest

The authors declare that they have no known competing financial interests or personal relationships that could have appeared to influence the work reported in this paper.
